# Prevalence and diversity of small rodent-associated *Bartonella* species in Shangdang Basin, China

**DOI:** 10.1371/journal.pntd.0010446

**Published:** 2022-06-01

**Authors:** Juan Yu, Bing Xie, Ge-Yue Bi, Hui-Hui Zuo, Xia-Yan Du, Li-Fang Bi, Dong-Mei Li, Hua-Xiang Rao

**Affiliations:** 1 Department of Basic Medical Sciences, Changzhi Medical College, Changzhi, China; 2 Department of Nursing, Changzhi Medical College, Changzhi, China; 3 Department of Clinical Medicine, Changzhi Medical College, Changzhi, China; 4 Department of Teaching and Scientific Research, Heping Hospital Affiliated to Changzhi Medical College, Changzhi, China; 5 State Key Laboratory for Infectious Disease Prevention and Control, Collaborative Innovation Center for Diagnosis and Treatment of Infectious Diseases, National Institute for Communicable Disease Control and Prevention, Chinese Center for Disease Control and Prevention, Beijing, China; 6 Department of Public Health and Preventive Medicine, Changzhi Medical College, Changzhi, China; Foshan University, CHINA

## Abstract

The aim of this study was to investigate the occurrence and molecular characteristics of *Bartonella* infections in small rodents in the Shangdang Basin, China. Small rodents were captured using snap traps, and their liver, spleen, and kidney tissues were harvested for *Bartonella* detection and identification using a combination of real-time PCR of the *ssrA* gene (296 bp) and conventional PCR and sequencing of the *gltA* gene (379 bp). Results showed that 55 of 147 small rodents to be positive for *Bartonella*, with a positivity rate of 37.41%, and 95% confidence interval of 29.50%- 45.33%. While the positivity rate across genders (42.62% in males and 33.72% in females, *χ*^2^ = 1.208, *P* = 0.272) and tissues (28.57% in liver, 33.59% in spleen, and 36.76% in kidney, *χ*^2^ = 2.197, *P* = 0.333) of small rodents was not statistically different, that in different habitats (5.13% in villages, 84.44% in forests, and 54.17% in farmlands, *χ*^2^ = 80.105, *P*<0.001) was statistically different. There were 42 *Bartonella* sequences identified in six species, including 30 *B*. *grahamii*, three *B*. *phoceensis*, two *B*. *japonica*, two *B*. *queenslandensis*, one *B*. *fuyuanensis* and four *unknown Bartonella* species from *Niviventer confucianus*, *Apodemus agrarius* and *Tscherskia triton*. In addition to habitat, *Bartonella* species infection could be affected by the rodent species as well. Among the *Bartonella* species detected in this area, *B*. *grahamii* was the dominant epidemic species (accounting for 71.43%). *B*. *grahamii* exhibited four distinct clusters, and showed a certain host specificity. In addition, 11 haplotypes of *B*. *grahamii* were observed using DNASP 6.12.03, among which nine haplotypes were novel. Overall, high occurrence and genetic diversity of *Bartonella* were observed among small rodents in the Shangdang Basin; this information could potentially help the prevention and control of rodent-*Bartonella* species in this area.

## Author summary

*Bartonella* species are recognized as emerging zoonotic pathogens that are mainly transmitted by blood-sucking arthropods, some being infectious to humans. Rodents are important natural reservoirs of *Bartonella* species. *Bartonella* infection has been detected in various countries worldwide, and their prevalence in rodents varies greatly across countries and regions. This is the first report of *Bartonella* prevalence and genetic diversity in Shangdang Basin of China. It revealed a high *Bartonella*-positivity rate in *Niviventer confucianus* and *Apodemus agrarius* in this area. Relatively higher occurrence of *Bartonella* species was detected in forests and farmlands. Six *Bartonella* species were detected, *B*. *grahamii*, *B*. *phoceensis*, *B*. *japonica*, *B*. *queenslandensis*, *B*. *fuyuanensis* and one *unknown Bartonella* species, of which *B*. *grahamii* was the dominant species, with high genetic diversity, and potentially pathogenicity in humans. Our study provided a better understanding of the occurrence and genetic diversity of *Bartonella* species in the small rodents in Shangdang Basin.

## Introduction

*Bartonella* species are newly discovered ancient gram-negative hemotrophic bacteria that are mainly transmitted by blood-sucking arthropods [1]. Over 40 species of *Bartonella* have been described till date, and they have a wide range of reservoirs, including cats, dogs, rodents, bats, carnivores and ruminants [2], among which rodents are considered important reservoirs. Previous studies had shown that the positivity rate of *Bartonella* to be 90.4% in *Onychomys torridus* in the United States, 69% in *Apodemus* in Japan, 17% in *Micromys minutus* in Russia, 78% in the rodents in Thailand, and 8.38%, 14.9%, 26.08%, and 57.7% in the rodents from eastern, southeastern, northwestern and northeastern of China respectively [3–8].

Humans can be infected via close contact with rodents, cats and dogs, or upon being bitten by blood-sucking arthropods, such as ticks, lice, fleas and mites [9], resulting in bartonellosis, including typical and common diseases, such as cat scratch disease and trench fever, and atypical diseases, such as neuroretinitis, arthritis, osteomyelitis, encephalitis, and bacteremia [10]. Till date, more than 10 *Bartonella* species have been recognized as human pathogens, such as *B*. *bacilliformis* [11], *B*. *quintana* [12], *B*. *henselae* [13], *B*. *elizabethae* [14], *B*. *clarridgeiae* [15], *B*. *koehlerae* [16], *B*. *vinsonii* subsp. *arupensis* [17], *B*. *vinsonii* subsp. *berkhoffii* [18], *B*. *grahamii* [19,20], *B*. *rochalimae* [21], *B*. *tamiae* [22], *B*. *ancashensis* [23], and *B*. *washoensis* [24]. Different species of *Bartonella* infection can cause human diseases with different clinical manifestations, being especially fatal in immunocompromised patients [25].

The Shangdang Basin is located southeast of Shanxi Province, China, between the Taihang and Taiyue Mountains, with an average elevation of 900–1000 m. It has a complex natural environment, with plains, hills, and mountains, and more than 10 species of rodents have been reported to inhabit this area since the 1990s, including but not limited to *Sciurotamias davidianus*, *Tamiops swinhoei*, *Apodemus agrarius*, *Apodemus peninsulae*, *Tamias sibiricus*, *Myospalax fontanieri*, *Tscherskia triton*, *Rattus norvegicus*, *Mus musculus*, *Cricetulus barabensis* [26]. Increased human activities have resulted in closer direct or indirect contact between humans and rodents, increasing the risk of transmission of rodent-associated *Bartonella* species. However, the *Bartonella* species in small rodents in this area have not yet been explored. This study aimed to investigate the occurrence and genetic diversity of *Bartonella* species in small rodents in Shangdang Basin. Our findings provided the insights into the distribution and genetic diversity of *Bartonella* in small rodents and presented a scientific basis for the control and prevention of *Bartonella* infection in humans in this area.

## Materials and methods

### Ethics statement

This study was approved by the Ethics Committee of Changzhi Medical College (No: DW2021052). All animals were treated according to the Guidelines of Regulations for the Administration of Laboratory Animals (Decree No. 2 of the State Science and Technology Commission of the People’s Republic of China, 1988) and the Guidelines for Treating Animals Kindly from Ministry of Science and Technology of the People’s Republic of China. All efforts were made to minimize discomfort to the animals.

### Rodents collection

**Fig 1:**
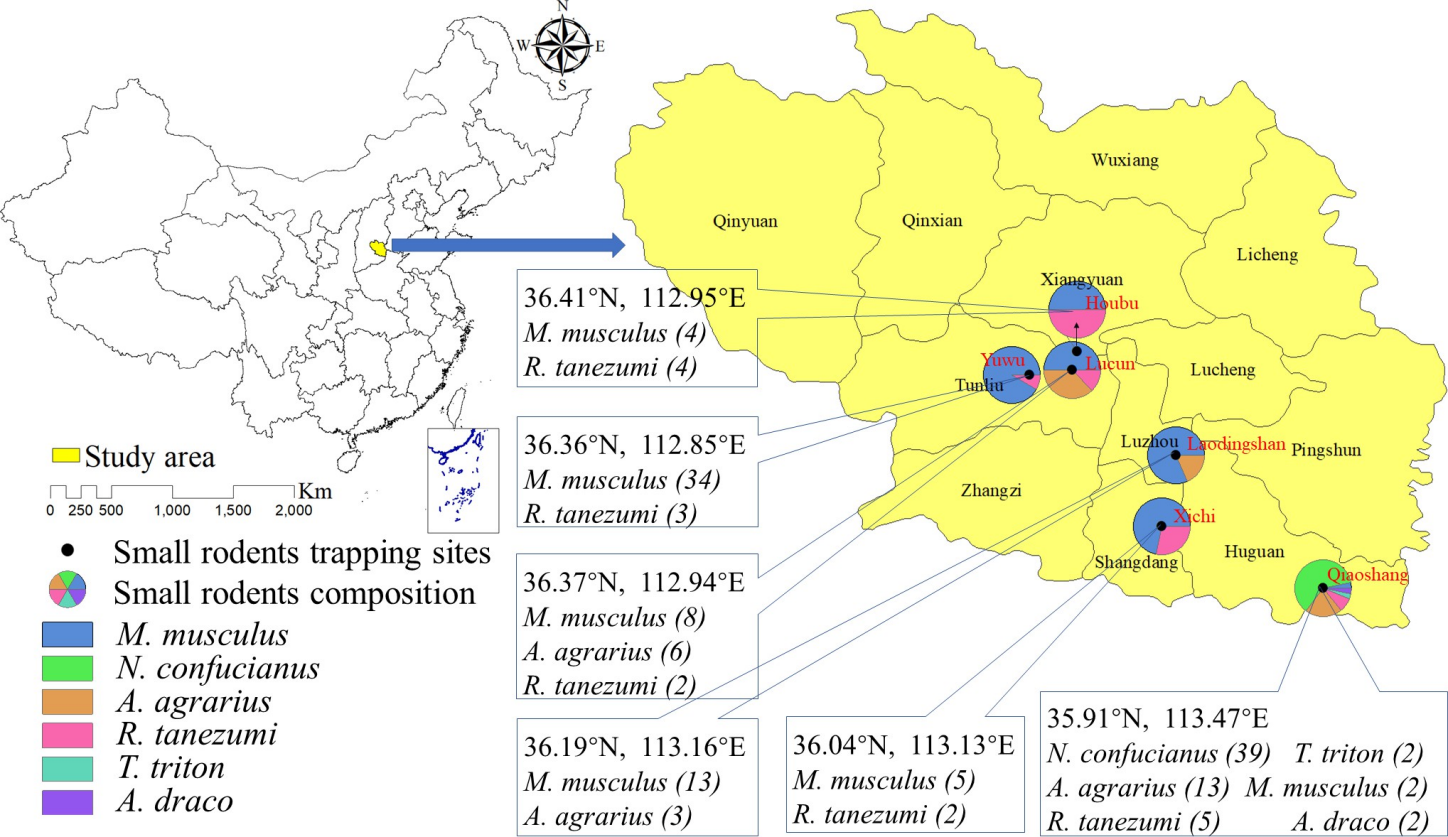
Geographical distribution of the trapped small rodents in the Shangdang Basin, China. The map was prepared in ArcGIS 10.2.2 using political boundaries from the National Geomatics Center of China (http://www.ngcc.cn/ngcc) for illustrative purposes only, these data are available free of charge.

Small rodents in Shangdang Basin (35.82°~37.12° N, 111.98°~113.73° E) of Shanxi Province were captured using snap traps in July 2020. Six rodent sampling sites from Luzhou District, Shangdang District, Huguan County, Xiangyuan County, and Tunliu District were randomly selected, and their geographical distribution is shown in Fig 1. Trapped rodents were identified by morphology and DNA barcoding based on the cytochrome C oxidase subunit I (CO I) gene. Liver, spleen and kidney tissues were harvested under sterile conditions from each rodent after euthanasia, and stored at -80°C until further use.

### *Bartonella* detection

DNA was extracted from approximately 10 mg of each liver, spleen and kidney tissue according to the manufacture’s protocols of TIANamp Micro DNA Kit (TIANGEN Biotech (Beijing) Co., Ltd., China). Real-time PCR was performed to detect the *Bartonella* by targeting a fragment of 296 bp of the transfer-mRNA (*ssrA)* gene according to a previous study [27]. Briefly, DNA amplification was performed in 20 μL mixtures containing 10 μL of HR qPCR Master Mix (Shanghai Huirui Bio-Tech Co., Ltd., Shanghai, China), 5 μL of double-distilled H_2_O, 0.8 μL (10 μmol/L) of each primer and 0.4 μL (10 μmol/L) probe (s*srA*-F: GCTATGGTAATAA ATGGACAATGAAATAA; *ssrA*-R: GCTTCTGTTGCCAGGTG; *ssrA*-P: FAM-ACCCCGCTTAAACCTGCGACG-BHQ1) [28], and 3 μL of DNA template. The amplification reaction mix was added to PCR strip tubes (AXYGEN, USA) and PCR was performed using the StepOnePlus (Applied Biosystems) under the following conditions: one cycle for 5 min at 95°C; 40 cycles for 15 s at 95°C, 45 s at 60°C, and positive and negative controls were set. Samples with Cq ≤ 35 were considered positive for amplification.

### *Bartonella* sequencing

For *ssrA-*positive samples, the *Bartonella* citrate synthase (*gltA*) gene amplification was further performed according to the manufacture’s protocols of TaKaRa PCR Amplification Kit (Takara Bio Inc., Japan), with 20 μL reaction mixtures containing 2 μL of 10 × PCR buffer, 1.6 μL of dNTP mix, 0.1 μL of Taq, 13.5 μL of double-distilled H_2_O, 0.4 μL (10 μmol/L) of each primer (BhCS781.p: GGGGACCAGCTCATGGTGG; BhCS1137.n: AATGCAAAAAGAACAGTAAACA) [29], and 2 μL of DNA template. *gltA* amplification was performed under the following conditions: one cycle for 5 min at 94°C; 35 cycles for 30 s at 94°C, 30 s at 55°C, and 60 s at 72°C; and a final extension for 10 min at 72°C. Next, PCR products with 379 bp were identified by 1.5% agarose gel electrophoresis, and then sent to Shanghai BioGerm Medical Technology Co., Ltd (Shanghai, China) for sequencing. Briefly, the purified PCR products were sequenced using an ABI Prism dye terminator cycle sequencing ready reaction kit and an ABI PRISM 3730XL DNA sequencer (Applied Biosystems, USA).

### Phylogenetic analysis

The sequences generated in this study were submitted to the GenBank (accession numbers: MZ672212-MZ672248, ON207132-ON207136). The nucleotide sequence homology was blasted against reported *Bartonella* species sequences in the GenBank using the BLAST program at the National Center for Biotechnology Information website (http://blast.ncbi.nlm.nih.gov/Blast.cgi). Phylogenetic tree was created using the maximum-likelihood method with MEGA version 7.0, and bootstrap values were calculated with 1000 replicates [30,31]. *Brucella abortus* was used as the outgroup.

### Genetic diversity analysis

*Bartonella-*positive sequences were analyzed for polymorphism based on the number of polymorphic sites (S), the number of haplotypes (H), nucleotide diversity (*π*), average number of nucleotide differences (*κ*) and haplotype diversity (Hd) using DNASP 6.12.03. Then, the sequences were analyzed based on a median-joining network using the Population Analysis with Reticulate Trees (PopART) software version 1.7 (http://popart.otago.ac.nz/index.shtml) with the default setting (epsilon = 0). *Bartonella* strains were downloaded from the GenBank, and their accession numbers are listed in S1 Table in the supplemental material.

### Statistical analysis

The positivity rates of *Bartonella* in different tissues, habitats and genders of small rodents were analyzed using the Chi-square test. Multivariate logistic regression analysis was performed to explore whether risk factors were associated with the occurrence of *Bartonella* species in rodents (*Bartonella* DNA positive *vs Bartonella* DNA negative). Odds ratios (ORs) and 95% confidence intervals were calculated to determine the strength of the associations. All data were analyzed using SPSS 22.0 (SPSS, Inc., Chicago, IL, USA). Statistical significance was set at *P* < 0.05.

## Results

### Rodents collection

In total, 147 trapped small rodents were identified into six species by morphology, including *Mus musculus* (66), *Niviventer confucianus* (39), *Apodemus agrarius* (22), *Rattus tanezumi* (16), *Tscherskia triton* (2), and *Apodemus draco* (2). Thereafter, rodents from different classifications were selected and their kidney tissues used for CO I gene sequencing. Since *M*. *musculus*, *N*. *confucianus*, and *A*. *agrarius* are easy to identify by morphology, we selected some of the samples for CO I gene sequencing. On the other hand, all rodents from *R*. *tanezumi*, *T*. *triton*, and *A*. *draco* species were confirmed by CO I gene sequencing. Sequencing results were consistent with the morphological classification. Geographical distribution of the trapped rodents was shown in Fig 1.

### Positivity of *Bartonella* species

Liver, spleen and kidney tissues were collected and used for *Bartonella* detection by real-time PCR amplification of the *ssrA* gene and cPCR of the *gltA* gene, both of them being positive in at least one tissue was considered positive for *Bartonella*. In total, 55 small rodents were positive for *Bartonella*, with a positivity rate of 37.41% (55/147, 95% CI: 29.50–45.33%), and were classified into four species (*N*. *confucianus* (34/39), *A*. *agrarius* (18/22), *T*. *triton* (2/2), and *A*. *draco* (1/2)). After deleting the missing specimens, 147 liver, 128 spleen, and 136 kidney specimens were used for further detection. The positivity rates in liver, spleen, and kidney were 28.57% (42/147, 95% CI: 21.18–35.96%), 33.59% (43/128, 95% CI: 25.30–41.89%), and 36.76% (50/136, 95% CI: 28.56–44.97%), respectively, and the difference in positivity rate in different tissues was not statistically significant (*χ*^2^ = 2.197, *P* = 0.333) (Table 1).

**Table 1:** Positivity rate of *Bartonella* infection in different tissues of small rodents.

Host	Liver		Spleen		Kidney		Total	
	No. detection	No. PCR positive (%)	No. detection	No. PCR positive (%)	No. detection	No. PCR positive (%)	No. captured	No. PCR positive (%)
MM	66	0 (0.00)	59	0 (0.00)	59	0 (0.00)	66	0 (0.00)
NC	39	24 (61.54)	35	26 (74.29)	36	31 (86.11)	39	34 (87.18)
AA	22	16 (72.73)	19	14 (73.68)	22	17 (77.27)	22	18 (81.82)
RT	16	0 (0.00)	11	0 (0.00)	15	0 (0.00)	16	0 (0.00)
TT	2	2 (100.00)	2	2 (100.00)	2	2 (100.00)	2	2 (100.00)
AD	2	0 (0.00)	2	1 (50.00)	2	0 (0.00)	2	1 (50.00)
Total	147	42 (28.57)	128	43 (33.59)	136	50 (36.76)	147	55 (37.41)

Abbreviations: MM: *Mus musculus*, NC: *Niviventer confucianus*, AA: *Apodemus agrarius*, RT: *Rattus tanezumi*, TT: *Tscherskia triton*, AD: *Apodemus draco*.

Of the 147 small rodents, 61 were male and 86 were female, and the positivity rate was 42.62% (26/61, 95% CI: 29.85–55.39%) in males and 33.72% (29/86, 95% CI: 23.53–43.92%) in females; the difference was not statistically significant (*χ*^2^ = 1.208, *P* = 0.272). Seventy eight small rodents of four species were captured in villages, with a *Bartonella* positivity rate of 5.13% (4/78, 95% CI: 0.12–10.13%). Forty five small rodents of five species were captured in forests, with a positivity rate of 84.44% (38/45, 95% CI: 73.43–95.46%). Twenty four small rodents of four species were captured in farmlands, with a positivity rate of 54.17% (13/24, 95% CI: 32.67–75.66%). Thus, the positive rates in different habitats were significantly different (*χ*^2^ = 80.105, *P* < 0.001) (Table 2). The multivariate logistic regression analysis showed that the infection risk in the farmland was 21.57 times that in the village (95% CI: 5.95–78.16), whereas infection risk in the forest was 86.69 times that in the village (95% CI: 24.52–306.42).

**Table 2:** Positivity rate of *Bartonella* infection of small rodents in different habitats.

Habitats	Host						No. captured	No. PCR positive	Positive rate (95% CI, %)	*b*	SE	*P*-value	OR (95% CI)
	MM	NC	AA	RT	TT	AD							
Village	57	5	0	15	0	1	78	4	5.13 (0.12–10.13)	-	-	-	-
Farmland	8	6	9	1	0	0	24	13	54.17 (32.67–75.66)	3.07	0.66	<0.001	21.57 (5.95–78.16)
Forest	1	28	13	0	2	1	45	38	84.44 (73.43–95.46)	4.46	0.64	<0.001	86.69 (24.52–306.42)

Abbreviations: CI: confidence interval, Reference group: Village

### Identifications of *Bartonella* species and distribution in rodents

**Fig 2:**
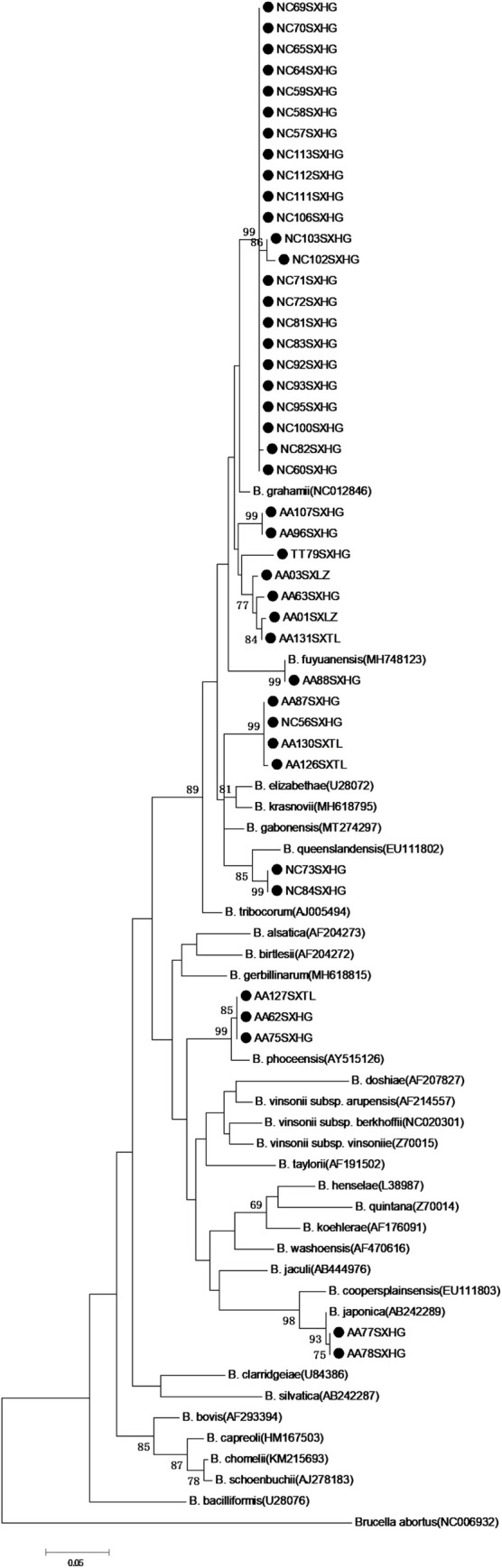
Phylogenetic tree constructed based on partial *gltA* gene (326 bp) of 42 *Bartonella* sequences. The tree was constructed by using the maximum-likelihood (ML) method with the Kimura 2-parameter model, bootstrap values calculated with 1000 replicates. The sequences detected in this study are indicated with black dots (NC represents *Niviventer confucianus*, AA represents *Apodemus agrarius* and TT represents *Tscherskia triton*). *Brucella abortus* was used as outgroup.

**Fig 3:**
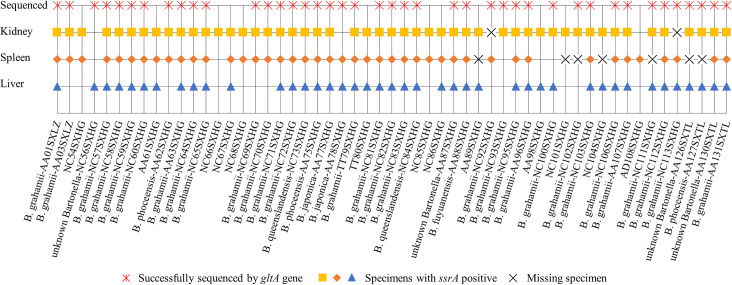
Detection of *Bartonella* species in various tissues of naturally infected rodents.

Sixty partial *gltA* sequences were obtained from 42 *Bartonella-*positive small rodents. DNA sequence homology analysis and phylogenetic analysis of the *gltA* gene indicated that six *Bartonella* species were detected in the liver, spleen, and kidney of small rodents, and that the *Bartonella* species detected in different tissues of each small rodent were consistent. Thirty sequences were shown to be *B*. *grahamii* with 96.05–100% identity, including 23 from *N*. *confucianus* (with highest identity of 97.30% with *Myodes rutilus* in China (KJ175044)), 6 from *A*. *agrarius* (including 4 sequences with highest identity of 99.08% with *A*. *agrarius* in northern China (KJ175032) and two sequences with highest identity of 100% with *A*. *agrarius* in southern China (EU179229)), and one from *T*. *triton* (with highest identity of 98.68% with *T*. *triton* in China (MH748115)); three sequences from *A*. *agrarius* were *B*. *phoceensis* with 96.86–99.74% identity (with highest identity of 99.74% with *A*. *agrarius* in China (KX549997)); two sequences from *A*. *agrarius* were *B*. *japonica* with 99.70% identity (with *Apodemus argenteus* in Japan (AB242289)); two sequences from *N*. *confucianus* were *B*. *queenslandensis* with 99.47–99.48% identity (with highest identity of 99.48% with *N*. *confucianus* in China (MH748120)); one sequence from *A*. *agrarius* was *B. fuyuanensis* with highest identity of 98.93% with *A*. *agrarius* in China (MH748123); three sequences of AA87SXHG, AA126SXTL and AA130SXTL from *A*. *agrarius* and one sequence of NC56SXHG from *N*. *confucianus* were *unknown Bartonella* species, which shared 94.23–95.26% nucleotide sequence similarity in their *gltA* fragment with the nearest species of *Bartonella*, *B*. *krasnovii* (with highest identity of 95.26% with *Flea* in Israel (CP031844)), *B*. *gabonensis* (with highest identity of 95.25% with *Lophuromys sp*. in Gabon (MT274297)) and *B*. *elizabethae* (with highest identity of 95.24% with *Meriones libycus* in Georgia (KT327032)), respectively (Figs 2 and 3).

**Fig 4:**
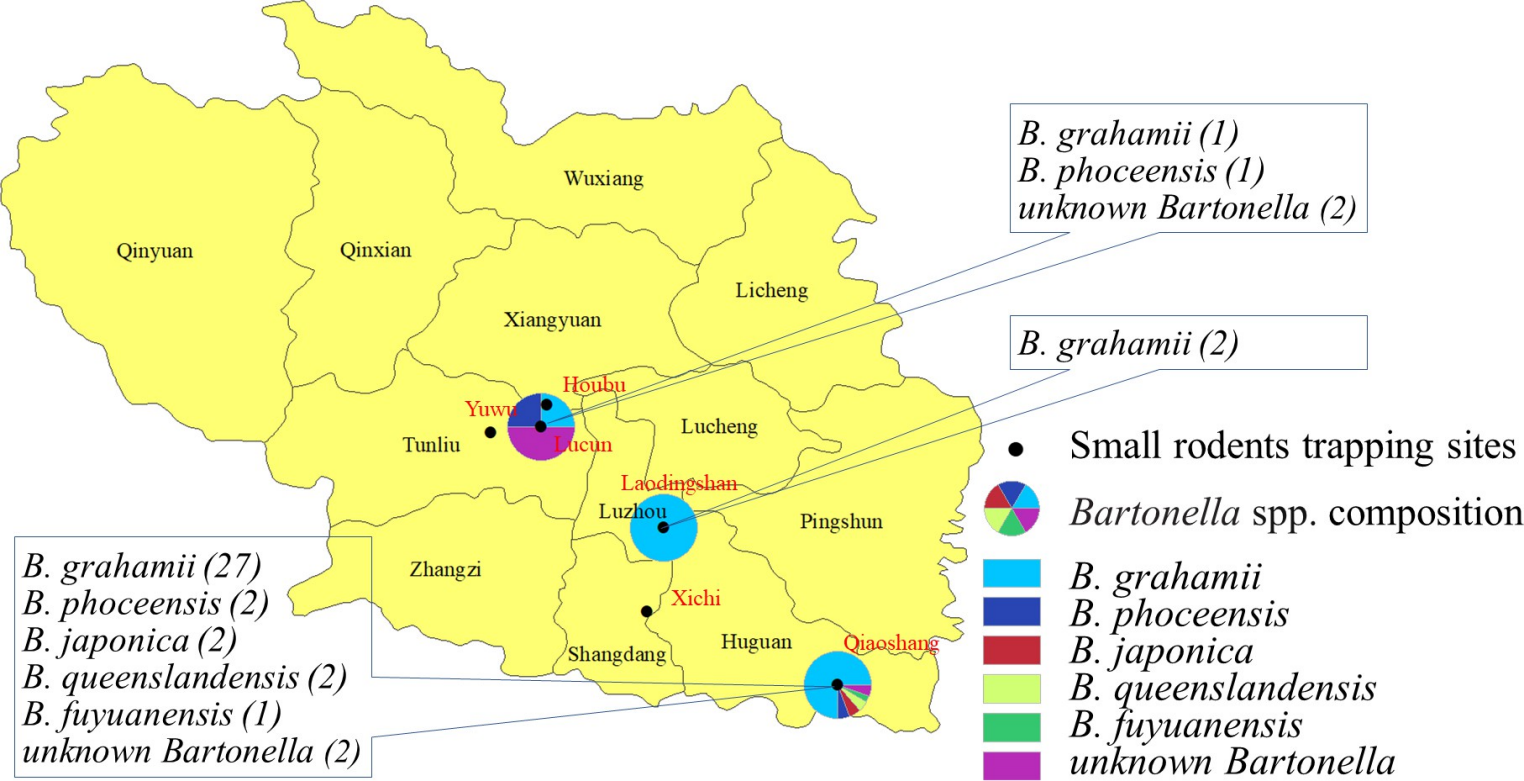
*Bartonella* species composition in different sampling sites in the Shangdang Basin, China. The map was prepared in ArcGIS 10.2.2 using political boundaries from the National Geomatics Center of China (http://www.ngcc.cn/ngcc) for illustrative purposes only, these data are available free of charge.

In this study, 26 *Bartonella* sequences of three different species were obtained from *N*. *confucianus*, including 23 of *B*. *grahamii*, two of *B*. *queenslandensis* and one of *unknown Bartonella* species; 15 *Bartonella* sequences of five different species were obtained from *A*. *agrarius*, including six of *B*. *grahamii*, three of *B*. *phoceensis*, two of *B*. *japonica*, one of *B*. *fuyuanensis*, and three of *unknown Bartonella* species; one *B*. *grahamii* sequence was obtained from *T*. *triton* and no *Bartonella* was detected in *M*. *musculus* and *R*. *tanezumi* (Table 3). In addition, *Bartonella* was detected in the small rodents from three of the six trapping sites, and the distribution of *Bartonella* species showed geographical differences (Fig 4).

**Table 3:** Distribution of *Bartonella* species in the infected small rodents.

Host	*B*. *grahamii*	*B*. *phoceensis*	*B*. *japonica*	*B*. *queenslandensis*	*B*. *fuyuanensis*	*Unknown Bartonella*	Total
MM	0	0	0	0	0	0	0
NC	23	0	0	2	0	1	26
AA	6	3	2	0	1	3	15
RT	0	0	0	0	0	0	0
TT	1	0	0	0	0	0	1
AD	0	0	0	0	0	0	0
Total	30	3	2	2	1	4	42

### Genetic diversity analysis

**Fig 5:**
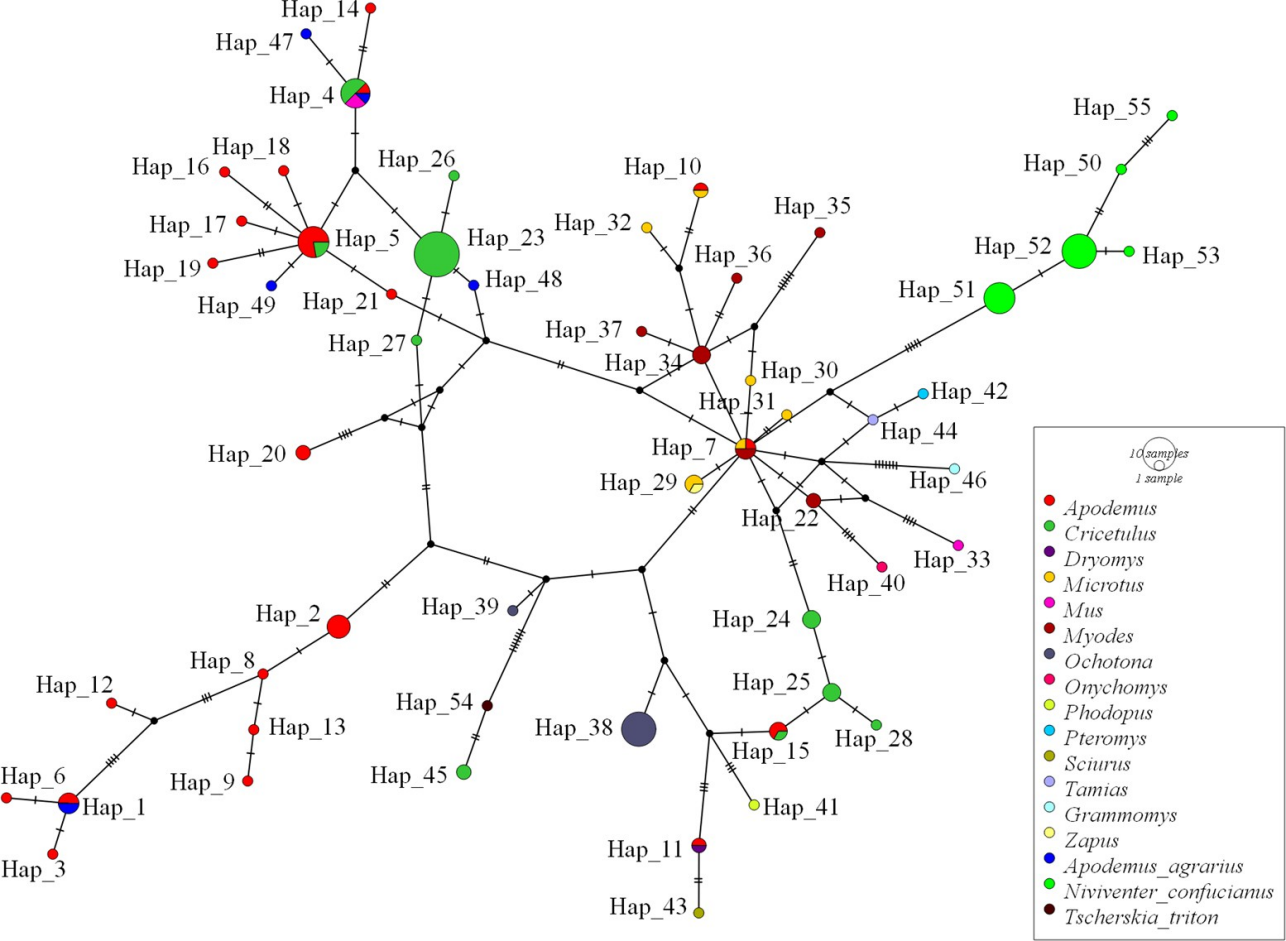
Median-joining networks of *gltA* gene for *B*. *grahamii* sequences from Shangdang Basin and other *B*. *grahamii* isolates from different rodents and regions. The sequences were analyzed based on a median-joining network using the Population Analysis with Reticulate Trees (PopART) software version 1.7 (http://popart.otago.ac.nz/index.shtml) with the default setting (epsilon = 0).

Genetic polymorphism analysis based on the *gltA* gene sequence (326 bp) showed 17 haplotypes in *Bartonella* species in this area (Hd = 0.884 *±* 0.033, *π* = 0.05306 *±* 0.00818), among which *B*. *grahamii* exhibited 11 haplotypes (Hd = 0.789 *±* 0.055, *π* = 0.01853 *±* 0.00398), *unknown Bartonella* had two haplotypes (Hd = 0.550 *±* 0.265, *π* = 0.00153 *±* 0.00081), and *B*. *phoceensis*, *B*. *japonica*, *B*. *queenslandensis* had one haplotype respectively (Table 4). We next constructed a haplotype network by comparing the sequences in our study with those of other *B*. *grahamii* strains isolated from different rodents and regions. Haplotype network analysis showed that six *B*. *grahamii* sequences from *A*. *agrarius* contained five haplotypes (two sequences (AA107SXHG and AA96SXHG) for Hap 1, and four sequences (AA131SXTL, AA01SXLZ, AA03SXLZ, and AA63SXHG) for Hap 4, 47, 48, and 49 respectively), which might be associated with the strains isolated from *Apodemus* spp. from China, Japan, South Korea, and the Russian Far East and from *Cricetulus longicaudatus* in China. Twenty three *B*. *grahamii* sequences from *N*. *confucianus* contained five haplotypes (Hap 50–53, 55), which clustered separately, and might be associated with the strains isolated from *Myodes rutilus* and *Microtus fortis* in China and from *Apodemus agrarius* in the Russian Far East. One *B*. *grahamii* sequence (TT79SXHG) from *T*. *triton* might be associated with a strain isolated from *T*. *triton* in China (Fig 5).

**Table 4:** DNA polymorphism of rodent-associated *Bartonella* species detected in Shangdang Basin.

*Bartonella* species (no. of sequences)	S	H	*κ*	Hd (mean ± SD)	*π* (mean ± SD)
*B*. *grahamii* (30)	31	11	6.041	0.789 ± 0.055	0.01853 ± 0.00398
*B*. *phoceensis* (3)	0	1	0.000	0.000	0.00000
*B*. *japonica* (2)	0	1	0.000	0.000	0.00000
*B*. *queenslandensis* (2)	0	1	0.000	0.000	0.00000
*unknown Bartonella* (4)	1	2	0.500	0.500 ± 0.265	0.00153 ± 0.00081
All sequences in this study (42)	85	17	17.298	0.884 ± 0.033	0.05306 ± 0.00818

Abbreviations: S, number of polymorphic sites; H, number of haplotypes; κ, average number of nucleotide differences; Hd, haplotype diversity; π, nucleotide diversity.

Note: *B*. *fuyuanensis* only had one sequence, can’t compute the DNA polymorphism.

## Discussion

Since the first isolation of *Bartonella* in 1993 from patients with HIV [32], studies on *Bartonella* have been carried out successively in various countries around the world, including the United States, Europe, Asia, Africa, Latin America and Oceania. Infection rate of *Bartonella* in rodents has been reported to be 6–90% in the United States [33,34], 60–83% in Russia [35], 4–57.7% in China [8,36,37], 6–94% in Japan [38,39], 7–14% in Korea [40], 13–63% in South Africa [41,42], 19% in Brazil [43], and so on. This indicated that the prevalence of *Bartonella* in rodents varies greatly across countries and regions; therefore, investigation of rodent-associated *Bartonella* infection in different areas would be important.

In this study, we observed the occurrence and molecular characteristics of *Bartonella* species in small rodents in the Shangdang Basin. To the best of our knowledge, this is the first report of its kind from this area. *Bartonella* culture is the gold standard for the detection and identification of *Bartonella* species; however, they are fastidious, slow growing and facultative intracellular bacteria, and it is difficult and time-consuming to culture them. At present, the fastest and most practical identification method is PCR identification of *gltA* [29], *rpoB* [44], ITS [45], *ribC* [46], *ftsZ* [47], and *ssrA* [28] genes. In our previous study, phylogenetic trees of *Bartonella* species were constructed based on the DNA sequences of *gltA*, *ftsZ*, *rpoB* and *ribC* genes respectively, and we had obtained the consistent results of *Bartonella* identification from these four genes [48]. The *gltA* gene is the most commonly used in the detection and classification of *Bartonella*, and *ssrA* gene has the same species identification function as *gltA* [28]. In this study, real-time PCR of *ssrA* gene and conventional PCR and sequencing of *gltA* gene were used in combination to detect and identify the *Bartonella* species.

Generally, spleen tissue is used for *Bartonella* detection. In this study, liver, spleen, and kidney tissues were used in combination for *Bartonella* detection, and the positivity rate was not significantly different across the tissues. However, multi-tissue detection could increase the overall positivity rate and obtained as many sequences as possible. The positivity rate of *Bartonella* species in small rodents was 37.41%, which was higher than that in most areas of China [49]. *Bartonella* species were detected in four of the six small rodent species, including *N*. *confucianus* (87.18%), *A*. *agrarius* (81.82%), *T*. *triton* (100%) and *A*. *draco* (50%). Considering that only two *A*. *draco* and two *T*. *triton* were captured, the positivity rates of 50% and 100% might not be the real *Bartonella* prevalence rates in these rodents, and further studies might be required for validation. However, no *Bartonella* was detected in *M*. *musculus* and *R*. *tanezumi* in our study, in contrast to the reports of some previous studies, such as no infection in *M*. *musculus* but 15.51% infection in *R*. *tanezumi* in Fujian, China [6], 8.3% infection in *M*. *musculus* in Lithuania [50], and 49.2% in *R*. *tanezumi* in Vietnam [51]. It revealed that the positivity rate of *Bartonella* in the rodents inhabit the farmland and forest was higher, which was similar to that reported in a previous study [48]. The infection risks of *Bartonella* in the farmland and forest were obviously higher than that in the village, indicating that the risk of *Bartonella* infection is significantly increased when people are engaged in wild activities; therefore, improvement in risk awareness and taking corresponding preventive measures would be necessary.

Based on *gltA* gene sequencing, 60 sequences were obtained from 42 small rodents, and the *Bartonella* species detected in different tissues of each small rodent were found to be consistent. Therefore, we selected 42 sequences from 42 small rodents for further analyses. DNA sequence homology and phylogenetic analyses. indicated that six *Bartonella* species were detected in the small rodents, including *B*. *grahamii*, *B*. *phoceensis*, *B*. *japonica*, *B*. *queenslandensis*, *B*. *fuyuanensis* and *unknown Bartonella* species. In addition, three species of *Bartonella* (*B*. *grahamii*, *B*. *queenslandensis* and *unknown Bartonella*) were detected in *N*. *confucianus* and five species of *Bartonella* (*B*. *grahamii*, *B*. *phoceensis*, *B*. *japonica*, *B*. *fuyuanensis*, and *unknown Bartonella*) were detected in *A*. *agrarius*. The *Bartonella* species detected in *N*. *confucianus* and *A*. *agrarius* in the same habitat were not completely the same, *B*. *queenslandensis* was only detected in *N*. *confucianus*, *B*. *phoceensis* and *B*. *japonica* were only detected in *A*. *agrarius*, suggesting that in addition to habitat, *Bartonella* species infection could be affected by the rodent species as well, and had a certain host specificity, which was similar to that reported in previous studies [8,33,36,52].

*B*. *grahamii* has been reported in many hosts, including *A*. *agrarius* (AB529500), *Apodemus flavicollis* (EU014266), *T*. *triton* (MH748118), *Microtus pennsylvanicus* (MK984788), *Microtus ochrogaster* (AB426656), etc. Moreover, we had previously identified *B*. *grahamii* in *Ochotona curzoniae* (KT445929), *Cricetulus longicaudatus* (MT815312), and *Microtus oeconomus* (MT815315). Here, it indicated that the sequences of *B*. *grahamii* from *A*. *agrarius* were clustered with the strains from *A*. *agrarius*, the sequence from *T*. *triton* were similar to the strain from *T*. *triton*, and the sequences from *N*. *confucianus* gathered in one unique cluster, which showed a certain host specificity in this area. To the best of our knowledge, *B*. *grahamii* was the first detected in *N*. *confucianus* in China. Additionally, *B*. *grahamii* was identified from three sampling sites, accounting for 71.43% (30/42) of all *Bartonella* species, suggesting that it was the dominant *Bartonella* species in Shangdang Basin. *B*. *grahamii* has been defined as a human pathogen [19,20], implying that close direct or indirect contact between humans and rodents may increase the risk of transmission of *B*. *grahamii* and cause human disease in this area. Whether the pathogenicity of *B*. *grahamii* is different in different rodents would require further study. A previous study had suggested that one *Bartonella* species could be identified to validated *Bartonella* species when they shared ≥ 96% nucleotide sequence similarity of *gltA* sequences [53]. Till date, *B*. *heixiaziensis* and *B*. *fuyuanensis* have been considered as new species identified in China [5,8]. Interestingly, our study indicated that one *unknown Bartonella* species, which shared ≤ 96% nucleotide sequence similarity in their *gltA* fragment with that of the reported *Bartonella* species, might be considered potential novel *Bartonella* species.

A previous study revealed that the highest polymorphism was within *gltA* and *rpoB* when similar parameters were calculated for sequences of known *Bartonella* species [8]. Our study observed 11 of the 17 haplotypes of partial *gltA* sequences for *B*. *grahamii*. Polymorphic level (Hd = 0.789) and nucleotide diversity (*π* = 0.01853) of *B*. *grahamii* (30 sequences) in our study were slightly greater than that of *B*. *grahamii* isolates (Hd = 0.700, *π* = 0.01227, five strains) from the northeast China [8]. This suggested the high genetic diversity of *B*. *grahamii* in Shangdang Basin, which could be the result of accelerated evolution of *Bartonella* species in rodents [54]. To further explore the origin of *B*. *grahamii*, we constructed a haplotype network by comparing the sequences in our study with those of other *B*. *grahamii* strains isolated from different rodents and regions. Currently, 55 haplotypes of *B*. *grahamii* were observed among approximately 15 genera of rodents, which exhibited a complex network, suggesting the evolution of *B*. *grahamii* to be complex. Generally, sequences from *A*. *agrarius* and *T*. *triton* are mainly related to the Asian strains isolated from *Apodemus* and *Cricetulus*, whereas the sequences from *N*. *confucianus* are mainly related to the Chinese strains isolated from *Myodes* and *Microtus* and the Russian Far East strains isolated from *Apodemus*. Additionally, among the 11 haplotypes detected in this study, only the sequences of two haplotypes were the same as that of the known *B*. *grahamii*, the remaining nine haplotypes were novel, suggesting that *B*. *grahamii* evolved rapidly in this area.

Our study has some limitations. First, the sample sizes of *T*. *triton* and *A*. *draco* were too small; thus, sample size would need to be increased in future to understand the real prevalence of *Bartonella* species. Secondly, some tissue samples did not contain a high quantity of bacteria, and hence not all the PCR-positive samples were sequenced successfully. The sequencing success rate was 76.37% (42/55), was higher than 14.68% (16/109) and 17.61% (56/318) in Thailand and Lithuania, respectively, similar to 78.95% (45/57) in Tanzania [50,55,56], which would need optimization in future studies. Thirdly, there seemed to be one *unknown Bartonella* species based on *gltA* gene analysis, which did not cluster with any reported *Bartonella* species. However, we only sequenced 326 bp segments within the *gltA* gene, and whether these unclassified *Bartonella* species are indeed novel would require further validation.

In conclusion, six species of *Bartonella*, including *B. grahamii*, *B. phoceensis*, *B. japonica*, *B. queenslandensis*, *B. fuyuanensis* and unknown *Bartonella* were detected in three species of rodents, *N. confucianus*, *A. agrarius* and *T. triton* in Shangdang Basin. Wild rodents were found to be more susceptible to *Bartonella* than domestic rodents. *B. grahamii* was the dominant species and had high genetic diversity in this area; its pathogenicity would require further investigation. Our study investigated the occurrence and molecular characteristics of *Bartonella* species among small rodents in Shangdang Basin; the information could potentially benefit the prevention and control of rodent-*Bartonella* species in this area.

## Supporting information

S1 Table The haplotypes and NCBI GenBank accession numbers of the previously characterized strains of *B*. *grahamii*. (PDF)
