# Supplementary material for: Prevalence and diversity of small rodent-associated Bartonella species in Shangdang Basin, China
Source: PLoS Negl Trop Dis. 2022 Jun 1;16(6):e0010446. doi: 10.1371/journal.pntd.0010446 (PMC9159596; doi:10.1371/journal.pntd.0010446)
Supplement: S1 Table — (PDF) [file pntd.0010446.s001.pdf]

Table S1. The haplotypes and NCBI GenBank accession numbers of the previously characterized strains of *B. grahamii*

Haplotype	Host	Country	Accession number
Hap-01	*Apodemus agrarius*	South Korea	AB529500
Hap-01	*Apodemus agrarius*	China	EU179229
Hap-02	*Apodemus agrarius*	China	EU179230
Hap-02	*Apodemus agrarius*	China	EU179231
Hap-02	*Apodemus agrarius*	China	EU179233
Hap-02	*Apodemus agrarius*	China	EU179235
Hap-02	*Apodemus agrarius*	China	EU179236
Hap-03	*Apodemus agrarius*	China	EU179234
Hap-04	*Mus musculus*	China	MT821837
Hap-04	*Mus musculus*	China	MT821834
Hap-04	*Cricetulus longicaudatus*	China	MT821828
Hap-04	*Cricetulus longicaudatus*	China	MT821826
Hap-04	*Cricetulus longicaudatus*	China	MT821822
Hap-04	*Cricetulus longicaudatus*	China	MT821820
Hap-04	*Apodemus agrarius*	China	KJ175030
Hap-05	*Cricetulus longicaudatus*	China	MT821838
Hap-05	*Cricetulus longicaudatus*	China	MT821823
Hap-05	*Apodemus agrarius*	China	KJ175032
Hap-05	*Apodemus speciosus*	Japan	AB242284
Hap-05	*Apodemus speciosus*	Japan	AB242286
Hap-05	*Apodemus* spp.	Japan	AB259954
Hap-05	*Apodemus speciosus*	Japan	AB426652
Hap-05	*Apodemus agrarius*	Russian Far East	AY584855
Hap-05	*Apodemus agrarius*	Russian Far East	AY584856
Hap-06	*Apodemus agrarius*	China	EU179232
Hap-07	*Myodes rutilus*	China	KJ175044
Hap-07	*Myodes rutilus*	China	KJ175060
Hap-07	*Microtus fortis*	China	KJ175067
Hap-07	*Apodemus agrarius*	Russian Far East	AY584857
Hap-08	*Apodemus chevrieri*	China	AF391271
Hap-09	*Apodemus draco*	China	AF391278
Hap-10	*Microtus agresti*	Sweden	AF391789
Hap-10	*Apodemus flavicollis*	Greece	AY435102
Hap-11	*Dryomys nitendula*	Greece	AY435121
Hap-11	*Apodemus flavicollis*	Greece	AY435122
Hap-12	*Apodemus peninsulae*	China	AF391275
Hap-13	*Apodemus peninsulae*	China	AF391280
Hap-14	*Apodemus peninsulae*	Russian Far East	AY584854
Hap-15	*Apodemus speciosus*	China	MT821840
Hap-15	*Apodemus speciosus*	China	MT821832
Hap-15	*Cricetulus longicaudatus*	China	MT815308

Hap-16	*Apodemus speciosus*	Japan	AB290286
Hap-17	*Apodemus speciosus*	Japan	AB290287
Hap-18	*Apodemus speciosus*	Japan	AB290288
Hap-19	*Apodemus speciosus*	Japan	AB290289
Hap-20	*Apodemus* spp.	Japan	AB259955
Hap-20	*Apodemus speciosus*	Japan	AB426653
Hap-21	*Apodemus speciosus*	Japan	AB529469
Hap-22	*Myodes gapperi*	Canada	AB426654
Hap-22	*Clethrionomys gapperi*	Canada	AY587976
Hap-23	*Cricetulus longicaudatus*	China	MT815304
Hap-23	*Cricetulus longicaudatus*	China	MT815310
Hap-23	*Cricetulus longicaudatus*	China	MT815303
Hap-23	*Cricetulus longicaudatus*	China	MT815302
Hap-23	*Cricetulus longicaudatus*	China	MT815301
Hap-23	*Cricetulus longicaudatus*	China	MT815300
Hap-23	*Cricetulus longicaudatus*	China	MT815299
Hap-23	*Cricetulus longicaudatus*	China	MT815298
Hap-23	*Cricetulus longicaudatus*	China	MT815297
Hap-23	*Cricetulus longicaudatus*	China	MT815296
Hap-23	*Cricetulus longicaudatus*	China	MT815295
Hap-23	*Cricetulus longicaudatus*	China	MT815294
Hap-23	*Cricetulus longicaudatus*	China	MT815293
Hap-23	*Cricetulus longicaudatus*	China	MT815292
Hap-23	*Cricetulus longicaudatus*	China	MT815291
Hap-23	*Cricetulus longicaudatus*	China	MT815289
Hap-23	*Cricetulus longicaudatus*	China	MT815288
Hap-23	*Cricetulus longicaudatus*	China	MT815287
Hap-23	*Cricetulus longicaudatus*	China	MT815286
Hap-24	*Cricetulus longicaudatus*	China	MT815311
Hap-24	*Cricetulus longicaudatus*	China	MT815306
Hap-24	*Cricetulus longicaudatus*	China	MT815290
Hap-25	*Cricetulus longicaudatus*	China	MT815313
Hap-25	*Cricetulus longicaudatus*	China	MT815309
Hap-25	*Cricetulus longicaudatus*	China	MT815305
Hap-26	*Cricetulus longicaudatus*	China	MT815307
Hap-27	*Cricetulus longicaudatus*	China	MT815312
Hap-28	*Cricetulus longicaudatus*	China	MT815314
Hap-29	*Zapus hudsonius*	America	MK984789
Hap-29	*Microtus pennsylvanicus*	America	MK984788
Hap-29	*Microtus ochrogaster*	America	AB426655
Hap-30	*Microtus ochrogaster*	America	AB426656
Hap-31	*Microtus oeconomus*	China	MT815315
Hap-32	*Microtus oeconomus*	Poland	EU014267
Hap-33	*Mus musculus*	America	AF086637

Hap-34	*Myodes glareolus*	France	JQ694003
Hap-34	*Myodes glareolus*	France	JX846159
Hap-34	*Myodes glareolus*	France	JX846164
Hap-35	*Myodes glareolus*	France	JQ694016
Hap-36	*Myodes glareolus*	France	JX846173
Hap-37	*Myodes glareolus*	England	Z70016
Hap-38	*Ochotona curzoniae*	China	KT445929
Hap-38	*Ochotona curzoniae*	China	KT445928
Hap-38	*Ochotona curzoniae*	China	KT445927
Hap-38	*Ochotona curzoniae*	China	KT445926
Hap-38	*Ochotona curzoniae*	China	KT445925
Hap-38	*Ochotona curzoniae*	China	KT445924
Hap-38	*Ochotona curzoniae*	China	KT445923
Hap-38	*Ochotona curzoniae*	China	KT445920
Hap-38	*Ochotona curzoniae*	China	KT445917
Hap-38	*Ochotona curzoniae*	China	KT445916
Hap-38	*Ochotona curzoniae*	China	KT445915
Hap-39	*Ochotona curzoniae*	China	KT445918
Hap-40	*Onychomys leucogaster*	America	DQ357612
Hap-41	*Phodopus sungorus*	America	AF110311
Hap-42	*Pteromys volans*	China	AB444994
Hap-43	*Sciurus vulgaris*	Czech Republic	MZ089839
Hap-44	*Tamias sibiricus*	China	AB444993
Hap-45	*Tscherskia triton*	China	MH748115
Hap-45	*Tscherskia triton*	China	MH748114
Hap-46	*Grammomys sp.*	Africa	FJ851112
